# K^+^ Accumulation and Clearance in the Calyx Synaptic Cleft of Type I Mouse Vestibular Hair Cells

**DOI:** 10.1016/j.neuroscience.2019.11.028

**Published:** 2020-02-01

**Authors:** P. Spaiardi, E. Tavazzani, M. Manca, G. Russo, I. Prigioni, G. Biella, R. Giunta, S.L. Johnson, W. Marcotti, S. Masetto

**Affiliations:** a Department of Brain and Behavioral Sciences, University of Pavia, Pavia 27100, Italy; b Department of Biology and Biotechnology, University of Pavia, Pavia 27100, Italy; c Department of Biomedical Science, University of Sheffield, Sheffield S10 2TN, UK

**Keywords:** AF, accumulation factor, LJP, liquid junction potential, MET, mechano-transducer, TEA, tetraethylammonium, Type I hair cell, calyx, K^+^ channel, vestibular, synapse, patch-clamp

## Abstract

Vestibular organs of Amniotes contain two types of sensory cells, named Type I and Type II hair cells. While Type II hair cells are contacted by several small bouton nerve terminals, Type I hair cells receive a giant terminal, called a calyx, which encloses their basolateral membrane almost completely. Both hair cell types release glutamate, which depolarizes the afferent terminal by binding to AMPA post-synaptic receptors. However, there is evidence that non-vesicular signal transmission also occurs at the Type I hair cell-calyx synapse, possibly involving direct depolarization of the calyx by K^+^ exiting the hair cell. To better investigate this aspect, we performed whole-cell patch-clamp recordings from mouse Type I hair cells or their associated calyx. We found that [K^+^] in the calyceal synaptic cleft is elevated at rest relative to the interstitial (extracellular) solution and can increase or decrease during hair cell depolarization or repolarization, respectively. The change in [K^+^] was primarily driven by *G*_K,L_, the low-voltage-activated, non-inactivating K^+^ conductance specifically expressed by Type I hair cells. Simple diffusion of K^+^ between the cleft and the extracellular compartment appeared substantially restricted by the calyx *inner* membrane, with the ion channels and active transporters playing a crucial role in regulating intercellular [K^+^]. Calyx recordings were consistent with K^+^ leaving the synaptic cleft through postsynaptic voltage-gated K^+^ channels involving K_V_1 and K_V_7 subunits. The above scenario is consistent with direct depolarization *and* hyperpolarization of the calyx membrane potential by intercellular K^+^.

## Highlights

• There is evidence that non-vesicular transmission occurs at the vestibular Type I hair cell-calyx synapse.
• K^+^ concentration in the calyceal synaptic cleft can increase or decrease_._
• Calyx recordings are consistent with the expression of low-voltage-activated K^+^ channels at the calyx *inner* membrane.
• Data support direct modulation of calyx membrane potential by intercellular K^+^ concentration.

## Graphical abstract

**Figure:**
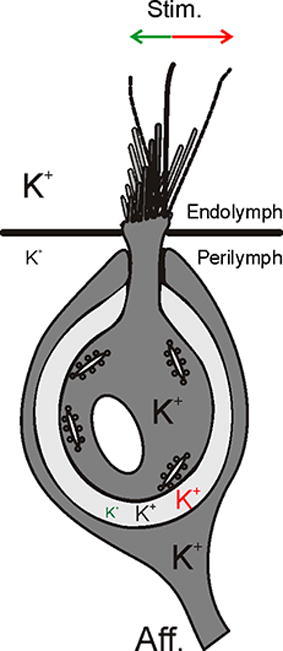

## Introduction

In mammalian, reptilian and avian species, head movements are detected by Type I and Type II vestibular hair cells. Only Type II hair cells are present in fish and amphibians. While the basolateral membrane of Type I hair cells is enclosed by a single giant afferent nerve terminal, called a calyx, each Type II hair cell is contacted by several (10 to 20) small bouton afferent endings and makes synapses with the outer faces of calyx endings (Lysakowski and Goldberg, 1997). Upon stimulation of vestibular hair cells, the opening of voltage-gated L-Type Ca^2+^ channels (Bao et al., 2003, Almanza et al., 2003, Zampini et al., 2006) triggers the exocytosis of glutamate, which depolarizes the afferent terminal by binding to AMPA receptors (Bonsacquet et al., 2006, Dulon et al., 2009, Songer and Eatock, 2013, Sadeghi et al., 2014, Kirk et al., 2017). However, a non-quantal mode of transmission has also been reported to occur at the Type I hair cell-calyx synapse (Yamashita and Ohmori, 1990, Holt et al., 2007, Songer and Eatock, 2013), though the molecular mechanism is not fully understood.

Given that the calyx confines a narrow (femtoliter) compartment that extends over a long distance, it has been speculated that K^+^ exiting the hair cell upon excitatory stimuli, accumulates rapidly in the synaptic cleft, thus directly depolarizing the pre- and postsynaptic membrane (Goldberg, 1996, Eatock and Lysakowski, 2006). Since the calyceal synaptic cleft is only a few tens of nm wide, direct measurement of the K^+^ concentration in the cleft has proved so far to be not possible. A recent study performed using dual whole-cell patch clamp in the turtle *crista* has shown that K^+^ efflux from the hair cell depolarizes the calyx, which is consistent with intercellular K^+^ accumulation (Contini et al., 2017).

Dual patch-clamp has not (yet) been achieved in mammalian vestibular epithelia, possibly because of the very thin and fragile neck region of the Type I hair cells. However, indirect information can be obtained by recording from Type I hair cells. Given that the basolateral membrane of Type I hair cells is completely enclosed in the afferent calyx, the patch pipette must be advanced through the calyx to reach it. Despite the calyx being pierced by the patch pipette, K^+^ efflux from the hair cell produces a shift of the K^+^ current reversal potential (*V*_rev_K^+^), consistent with K^+^ accumulation in the intercellular compartment enclosed by the residual calyx (Lim et al., 2011, Contini et al., 2012). Indeed, such a shift of *V*_rev_K^+^ was not observed while recording from mouse Type II hair cells (Contini et al., 2012).

The reported shift of *V*_rev_K^+^ (Lim et al., 2011, Contini et al., 2012) was highly variable among recordings, possibly because of different levels of damage produced to the calyx. The condition of the calyx after piercing is not visually assessable, precluding a correlation between residual calyx morphology and hair cell electrophysiology. Therefore, we first analyzed the dependence of *V*_rev_K^+^ on K^+^ current elicited in Type I hair cells by investigating the accumulation factor (AF). The AF provides an indication of the “quality” of the residual calyx in terms of its residual ability to confine intercellular K^+^. Large AF values correlated with a depolarized resting membrane potential of the hair cell and a pronounced outward K^+^ current relaxation. We also found that intercellular [K^+^] increased or decreased depending on the size, kinetics and direction of K^+^ flow through *G*_K,L_, the low-voltage activated K^+^ conductance specifically expressed by Type I hair cells. Finally, since *V*_rev_K^+^ also depends on postsynaptic K^+^ channels, we recorded from *in situ* calyces. Our results are consistent with K_V_1 and K_V_7 channels being involved in the direct modulation of the postsynaptic membrane by intercellular K^+^.

## Experimental procedures

### Ethical statement

All procedures for animal housing and experimentation were approved by the Ministero Italiano della Salute (Rome, Italy) and animal experiments were carried out in accordance with the European Communities Council Directive of 24 November 1986 (86/609/EEC). Mice (Swiss CD1 and C57BL/6N) from both sexes were obtained from Charles River (Italy) and from the Animal Care Facilities of the University of Pavia (Italy). In the UK, experiments were performed in accordance with Home Office regulations under the Animals (Scientific Procedures Act) 1986 and following approval by the University of Sheffield Ethical Review Committee. Mice were maintained under controlled light–dark cycles and received rodent pellets and water *ad libitum*.

### Cell preparation

The age of the mice ranged from postnatal day (PD) 7 to P47. Semicircular canal *crista* Type I hair cells were recorded either *in situ* or after enzymatic dissociation, as indicated in figure legends or text. A few recordings were also made *in situ* from mouse utricle Type I hair cells. All calyces were recorded *in situ* from the mouse *crista* or utricle. Data from utricle and canal hair cells were pooled. The location of the recorded cells was not assessed. Briefly, following anesthesia via inhalation with halothane (2-Bromo-2-Chloro-1,1,1-trifluoroethane, 99%; Sigma-Aldrich) in Italy, and cervical dislocation in the UK, mice were decapitated and *ampullae* or utricles were surgically removed in chilled extracellular solution (Extra_std, in mM): NaCl 135, CaCl_2_ 1.3, KCl 5.8, MgCl_2_ 0.9, HEPES 10, glucose 5.6, NaH_2_PO_4_ 0.7, Na-pyruvate 2. Vitamins (GIBCO Invitrogen, 10 mL/L) and amino acids (GIBCO Invitrogen, 20 mL/L) for Eagle’s minimum essential medium (MEM) were also added. The pH was adjusted to 7.4 with NaOH (final osmolality: 310 mOsm/kg).

For *in situ* recordings, following vestibular ganglia removal the *cristae* or the *maculae* were immobilized at the bottom of the recording chamber by mean of a weighted nylon mesh. Sensory epithelia were viewed by using an upright microscope equipped with differential interference contrast optics (Olympus or Nikon, Japan) and 60× water immersion objective. For hair cell dissociation, the mechanical-enzymatic treatment was the same as reported in Spaiardi et al. (2017).

Recordings were obtained from 76 Type I hair cells *in situ* and 44 isolated Type I hair cells with the K^+^-based intracellular solution (see below), from 17 *in situ* Type I hair cells with the Cs^+^-based intracellular solution (see below), and from 22 *in situ* calyces (20 with Intra_Cs^+^ and 2 with Intra_K^+^). In some experiments, the general outward rectifier K^+^ channels blockers tetraethylammonium (TEA, Fluka, Sigma-Aldrich) and 4-Aminopyridine (4-AP, Sigma-Aldrich), plus Cs^+^ which also blocks HCN channel (Biel et al., 2009), were added to the extracellular solution. The composition of the extracellular solution containing the above K^+^ channel blockers was as follows (in mM): NaCl 110, CaCl_2_ 1.3, CsCl 5.8, MgCl_2_ 0.9, HEPES 10, glucose 5.6, NaH_2_PO_4_ 0.7, TEACl 30, 4-AP 15. The pH was adjusted to 7.38 with HCl (final osmolality: 312 mOsm/kg). In some experiments, CdCl_2_ 0.1 mM (Sigma-Aldrich) was also added to the latter solution to block the Ca^2+^ current. All solutions were made freshly every morning and used in the course of the day.

### Patch-clamp whole-cell recordings

The amplifier’s filter bandwidth was generally set at 5 or 10 kHz. Digital sampling frequency was three to five times the analog bandwidth of the signal recorded. Current and voltage were measured and controlled through a DigiData 1322A or 1440 interface (AD/DA converter; Molecular Devices, USA) connected to a computer running pClamp software.

Whole-cell recordings from Type I hair cells *in situ* were obtained after removal of visible calyx and tissue debris above the hair cell by using the patch pipette, and the ‘cleaning’ procedure was repeated with one (or more) patch-pipette until a GigaΩ-seal was performed. For dissociated hair cells, removal of the residual calyx by the patch pipette was not possible because during the procedure the hair cells became detached from the bottom of the Petri dish.

For calyx recordings, after seal formation and suction, calyx identification was assessed by the presence of Na^+^ currents. Sometimes, the seal was performed in ‘blind patch’ because the thickness of the preparation precluded visual identification of the thin calyx structure.

Whole-cell recordings were obtained in voltage-clamp mode at room temperature (RT, 22–24 °C). A few recordings, as noted in the figure legends, were obtained at body temperature (BT, 35–37 °C). The patch-clamp amplifier was an Axopatch 200B (Molecular Devices, USA) or Optopatch (Cairn Research Ltd, UK) amplifier. Patch pipettes were pulled from soda glass capillaries (Hilgenberg, Germany), fire-polished (in some cases) and partially coated with Sylgard (Dow Corning 184, Midland, MI) or surf wax (Mr. Zogs SexWax, USA). The micropipettes were filled with a K^+^-based intracellular solution (Intra_K^+^; in mM): KCl 131, MgCl_2_ 3, Na_2_-Phosphocreatine 10, Na_2_ATP 5, HEPES 5, EGTA 1, pH 7.2 with KOH, for a final osmolality of 293 mOsm/kg. In some experiments, K^+^ was omitted from the pipette solution, which contained (Intra_Cs^+^; in mM): l-glutamic acid 110, CsCl 20, Na_2_-Phosphocreatine 10, MgCl_2_ 3, Na_2_ATP 5, Hepes 5, EGTA 1, GTP 0.3, pH 7.28 with CsOH, for a final osmolality of 290 mOsm/kg). When filled with either intra-pipette solution, micropipettes had a resistance in the bath of 2–5 MΩ. All voltages in text and figures were corrected for the liquid junction potential (LJP) of −4 mV when using Intra_K^+^ or −11 mV when using Intra_Cs^+^, measured between electrode and bath solution (Neher, 1992). All values in text and figures were corrected for LJP. Leakage was not subtracted except when noted in the text.

The cell resting membrane potential (*V*_rest_) was measured with the K^+^-based intracellular solution as the zero-current voltage in current-clamp mode.

For Type I hair cells, the membrane input resistance (*R*_m_) was measured in voltage-clamp from the steady-state current elicited by a voltage step from −64 mV, or −61 mV, to −54 mV, or −51 mV, in Intra_K^+^ or Intra_Cs^+^, respectively. Since *G*_K,L_ is fully active near −60 mV (Rennie and Correia, 1994, Rüsch and Eatock, 1996), *R*_m_ is mainly determined by *G*_K,L_. To compare leakage between cells, *R*_m_ was calculated between −124 mV and −114 mV, at which voltages *G*_K,L_ was fully deactivated. As far as the possible contribution from the hyperpolarization-activated mixed Na^+^/K^+^ current through HCN-channels (*I*_h_) is concerned, this current was only detected in a minority of Type I hair cells and, when present, it was very small (see Results). Series resistance (*R*_s_) and cell membrane capacitance (*C*_m_) were calculated off-line by the capacitive artifact elicited by a voltage step from −124 mV to −44 mV in Intra_K^+^, or −131 mV to −51 mV in Intra_Cs^+^. At these voltages *I*_K,L_ and *I*_K,v_ activated slowly enough to minimize overlap with the capacitive artefact. Moreover, a possible contamination by *I*_h_, when present, should be minimal since with Intra_K^+^ and Extra_std it should reverse close to −40 mV (Holt and Eatock, 1995). Different from the other voltage protocols, that used to generate the current transient for *C*_m_ and *R*_s_ measurement had a sampling rate of 100 kHz (an example is shown in the enclosed raw data file Neuroscience16226048Cm&Rs). Fit was performed from the average trace of 10 sweeps. We did not attempt on-line *R*_s_ compensation because *G*_K,L_ is active up to −100 mV and repetitive voltage pulses more negative than −100 mV applied in close sequence damaged the cell. Moreover, given the residual calyx, *R*_s_ might also include a small contribution from the intercellular resistance. Therefore, we preferred to minimize *R*_s_ by keeping the pipette resistance as low as possible (tip diameters of about 2 μm) despite the greater difficulty in obtaining a gigaΩ-seal. We calculated an average *R*_s_ of 8.13 ± 5.34 MΩ (*n* = 120). Given an average *C*_m_ of 9.36 ± 6.12 pF (*n* = 119), the mean voltage-clamp time constant of the amplifier calculated by multiplying *C*_m_ and *R*_s_ for each cell was 58 ± 30 μs (*n* = 119). Although the clamp speed was reasonably good, depolarization above −30 mV elicited K^+^ currents of several nA in Type I hair cells, thus producing large voltage drops across the residual *R*_s_ (*V*_Rs_). For the analysis of the relation between the quantity of K^+^ flowing through the cell membrane and tail current amplitude we did not correct for *V*_Rs_ since tail currents at −44 mV had a limited peak amplitude (0.62 ± 0.46nA; *n* = 119), producing a mean *V*_Rs_ of 5.19 ± 4.98 mV (*n* = 119). As far as the experiments with Intra_Cs^+^ are concerned, the amplitude of the currents, and therefore *V*_Rs,_ was much smaller than with Intra_K^+^ (∼one tenth, see Table 1) and, except for Fig. 6B, voltages were not corrected because *V*_Rs_ was <2 mV. In the other cases, as stated in the text, voltages were corrected for *V*_Rs_. All *R*_s_ values are provided in Figure legends.

**Table 1:** Different parameters for Type I hair cells recorded with Intra_K^+^ or Intra_Cs^+^. Values are shown as mean ± S.D. The peak outward current (*I*_p_) was measured at −4 mV or −1 mV with Intra_K^+^ or Intra_Cs^+^, respectively. The number of cells is shown in brackets

	*V*_rest_ (mV)	*R*_m_ (MΩ)	*I*_p_ (nA)	*V*_rev_ (mV)
Intra_K^+^	−72.2 ± 4.5 (115)	25.3 ± 15.2 (99)	4.19 ± 1.81 (115)	−74.4 ± 4.2 (115)
Intra_Cs^+^	N.A.	109.4 ± 75.3 (16)	0.66 ± 0.62 (16)	−40.0 ± 6.3 (17)

For calyx recordings, *R*_s_ and *C*_m_ were calculated in a similar way as for Type I hair cells. Since most calyces recorded with Intra_Cs^+^ (16 of 20) fired repetitively, indicative of inadequate space-clamp, *C*_m_ measurements were not considered. In the 4 remaining calyces, the mean *C*_m_ was 39.0 ± 18.1 pF (*n* = 4).

### Data analysis

Analysis of traces and results were performed with Clampfit (pClamp version 10, USA), Origin 6.1 (OriginLab., USA) and Microsoft Excel (Microsoft Corporation, USA). The quantity of K^+^ ions flowing during a given voltage step was calculated by integrating the macroscopic current tracing with Clampfit, which provided a quantity of charge ms^−1^ (Q).

The equilibrium potential for K^+^ (*E*_K_) was calculated according to the Nernst equation:

(1) $$E_{K}=RT/F\;\left(\text{ln}\left({\left[{\text{K}}^{+}\right]}_{\text{out}}/{\left[{\text{K}}^{+}\right]}_{\text{in}}\right)\right)$$

where the subscripts “out” and “in” refer to the extracellular and intracellular solution, respectively.

The macroscopic current reversal potential (*V*_rev_) of the mixed Cs^+^/K^+^ current (called a “biionic potential”, see Hille, 2001) for current through *G*_K,L_ was calculated according to the following equation:

(2) $$V_{\mathrm{rev}}=RT/F\left(\text{ln}\left(P_{\text{A}}{\left[{\text{A}}^{+}\right]}_{\text{out}}/{\text{P}}_{\text{B}}{\left[{\text{B}}^{+}\right]}_{\text{in}}\right)\right)$$

where *P* is the relative permeability of the ions A^+^ and B^+^.

*G*_K,L_ activation curve in Intra_Cs^+^ was generated by fitting the average normalized chord conductance, calculated by the current elicited at voltages from −111 mV to −51 mV (10 mV increment) delivered from the conditioning voltage of −131 mV and considering a *V*_rev_ of −40 mV (see Results), with the following Boltzmann function:

(3) $$G\left(V\right)=G_{\text{max}}+\left(G_{\text{min}}--G_{\text{max}}\right)/\left(1+\;\text{e}\left(V-V_{1/2}\right)/S\right)$$

where *G*(*V*) is conductance at voltage *V*, *G*_min_ and *G*_max_ are minimum and maximum chord conductances, *V*_1/2_ is voltage corresponding to half-maximal activation, and *S* is the voltage corresponding to an e-fold increase in *G*(*V*).

### Additional information

All raw data cited in results can be found in folders *NeuroscienceFig1* to 7, together with Origin files used for figures. All analyses performed can be found in *NeuroscienceDatasheetExcel*.

### Statistical methods

Statistical analysis was performed by Prism GraphPad 6.0 Software (San Diego, CA, USA). Following Kolmogorov-Smirnov normality test, Mann–Whitney, or unpaired *t*-test with or without Welch correction, was used for mean (median) comparison, as stated in the text. For parametric tests, the degrees of freedom and statistic’s values (*t* and *F*), in addition to the p value, are shown in the text. For non-parametric tests (Mann–Whitney), the statistic’s value U is provided in addition to the p value. Statistical relationship between two quantitative, continuous variables was estimated providing the Pearson’s correlation coefficient. Data are expressed as median and/or mean ± standard deviation (S.D.), or standard error (S.E.) when indicated; *n* = number of values.

In a few cases, recordings were obtained from distinct cells from the same animal, as follows. With Intra_K^+^ in the pipette, 74 recordings were obtained from 74 different mice, while 46 from 20 mice, of which 16 provided 2 recordings each, 1 provided 3 recordings, and 2 provided 4 recordings each. Therefore, the contribution from nested data was largely diluted in the data pool. Moreover, AF measurements obtained from cells of the same animal were not clustered (see *NeuroscienceDatasheetExcel*, worksheet: Nested data). As far as data used for AF statistical analyses is concerned, they all came from different mice except for 2 cells (in 10) coming from the same animal for the low-AF group, for which two cells the average value was taken. Therefore, we did not perform a multilayer analysis. Finally, as far as recordings with Intra_Cs^+^ are concerned, all averaged values are from different animals.

## Results

### Intercellular K^+^ accumulation

Type I hair cells distinctively express the large outward rectifier K^+^ conductance *G*_K,L_, which activates at about −100 mV, is almost fully active at −60 mV and shows negligible inactivation during hundreds ms depolarizing steps (Rennie and Correia, 1994, Rüsch and Eatock, 1996, Chen and Eatock, 2000, Hurley et al., 2006, Eatock and Songer, 2011). Another typical feature of *G*_K,L_ is that the voltage-dependence of its activation curve can differ significantly between cells, and even in a same cell during the whole-cell recording, presumably depending on the level of channel phosphorylation (Hurley et al., 2006).

In addition to *G*_K,L_, Type I hair cells express the small delayed outward rectifier K^+^ conductance *G*_K,v_, which activates near −40 mV and inactivates slowly (Rennie and Correia, 1994, Rüsch and Eatock, 1996, Eatock and Songer, 2011, Spaiardi et al., 2017). Finally, most Type I hair cells from the mouse utricle express the mixed cationic *h*-conductance (*G*_h_), which activates for hyperpolarization below −60 mV (Horwitz et al., 2011). However, *G*_h_ was rarely detected in our recordings from the mouse *crista*. It has been shown that K^+^ exiting through *G*_K,L_ and *G*_K,v_ can produce a significant (tens of mV) shift of the K^+^ current reversal potential (*V*_rev_K^+^) towards depolarized voltages, which has been attributed to K^+^ accumulation in the residual calyceal synaptic cleft (Lim et al., 2011, Contini et al., 2012). To better understand the nature and effects of the shift of *V*_rev_K^+^, we have recorded the whole-cell response from dissociated and *in situ* Type I hair cells and from the associated calyx.

**Fig. 1:**
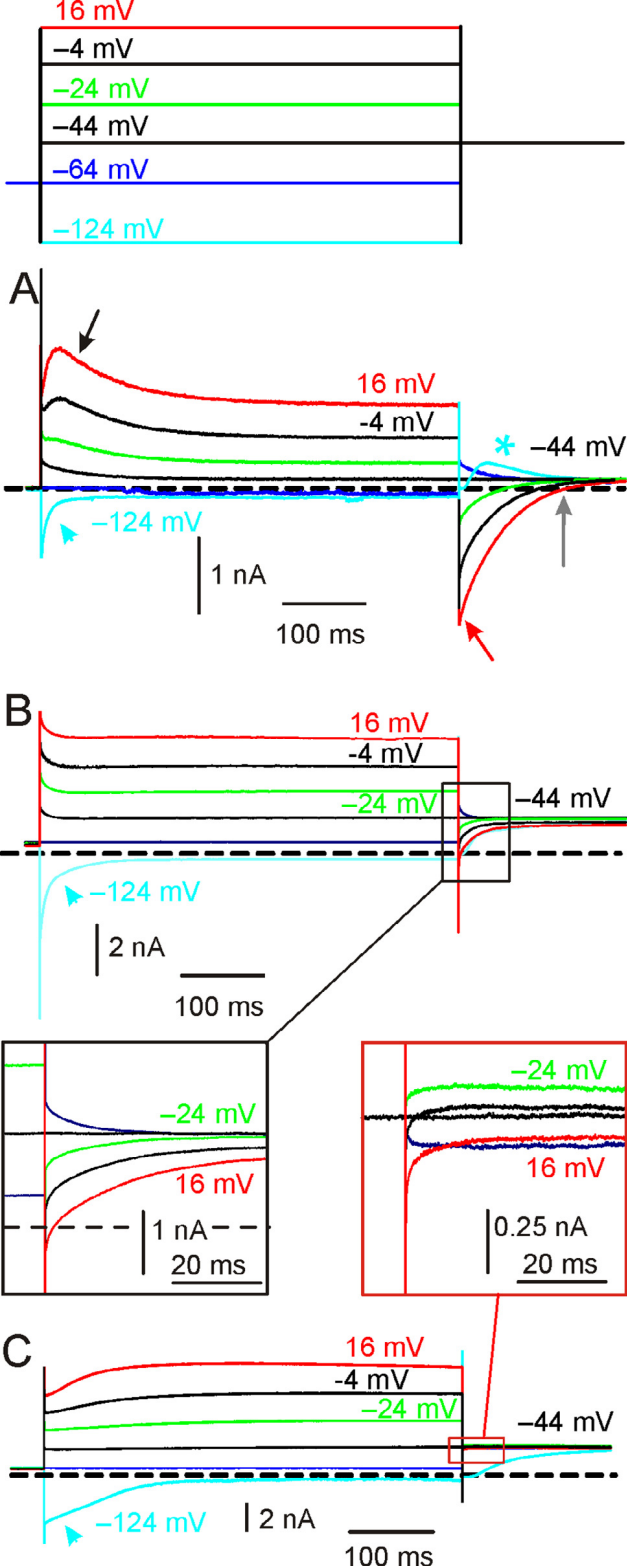
Whole-cell currents recorded from Type I hair cells. (**A**) Current response showing substantial K^+^ accumulation around the hair cell. Here and in the next figures, capacitive artefacts were partially blanked and the horizontal dashed line indicates the zero-current level. Currents were elicited by conditioning voltage steps (V_conds_) of 500 ms duration delivered from a holding potential (V_hold_) of −64 mV, as from the voltage protocol shown at the top. Since *G*_K,L_ is fully active at −60 mV, V_cond_ depolarization or hyperpolarization elicited an instantaneous outward or inward current. As far as the outward current is concerned, after the initial instantaneous component, it reached a peak and then decreased (black arrow). The decrease is due to K^+^ accumulation around the hair cell shifting *V*_rev_K^+^ toward more positive voltages (Contini et al., 2012). Following most depolarized V_conds_, repolarization to the test potential (V_test_) of −44 mV elicited an inward (red arrow) instantaneous tail current (*I_i_*__tail_). The inward current amplitude then decreased up to reverse (grey arrow). The time course of the inward current corresponds to the progressive shift of *V*_rev_K^+^ back toward more negative voltages. Upon V_cond_ hyperpolarization to −124 mV, an initial inward instantaneous current through *G*_K,L_ is produced, followed by its complete deactivation (the cyan arrowhead points at the decay time course). Upon depolarization to −44 mV *G*_K,L_ re-activates, although the activation time course cannot be properly appreciated because of progressive intercellular K^+^ accumulation, as obvious from its relaxation (asterisk). Dissociated hair cell, P18, RT. *R*_s_: 8.28 MΩ. File: 13531027. (**B**) Current response showing evidence of K^+^ accumulation, elicited in a different Type I hair cell. The outward current showed a clear relaxation although *I_i_*__tail_ showed a minor shift compared to A and only reversed following the most depolarized V_cond_ (see also inset in the black box below). Hair cell *in situ*, P7, RT. *R*_s_: 5.48 MΩ. File: 10n24000. (**C**): Current response showing little evidence of K^+^ accumulation, elicited in a different Type I hair cell. The outward current showed a very small relaxation only at the most depolarized V_cond_ and *I_i_*__tail_ showed a limited shift and was always outward even following the most depolarized V_cond_ (see also inset in the red box above). Also note that *G*_K,L_ re-activation at −44 mV following V_cond_ of −124 mV (cyan trace) does not show any relaxation, revealing the *G*_K,L_ activation time course. Hair cell *in situ*, P14, RT. *R*_s_: 3.01 MΩ. File: 13o16001. See folder *NeuroscienceFig1* for raw data and Origin files. (For interpretation of the references to colour in this figure legend, the reader is referred to the web version of this article.)

To facilitate the description of the following results, we provided three representative current responses in the presence of a very large (Fig. 1A), a limited (Fig. 1B), or a negligible (Fig. 1C) shift of *V*_rev_K^+^ (see also Contini et al., 2012). The best evidence for the shift of *V*_rev_K^+^ is the reversal of the instantaneous tail currents (*I*_i_tails_) at −44 mV (red arrow in Fig. 1A) following conditioning depolarizing voltage steps (V_conds_). Note that, in the absence of intercellular K^+^ accumulation, the outward *I*_i_tails_ should increase with V_cond_ depolarization, consistent with the increase of the K^+^ conductance and the driving force. When substantial intercellular K^+^ accumulation occurs, the outward *I*_i_tails_ will decrease with V_cond_ depolarization, because of the increase in K^+^ exit, until eventually reversing. The reversal of *I*_i_tails_ is associated with a relaxation of the outward K^+^ current during V_cond_ (black arrow in Fig. 1A), which is likely produced by the progressive shift of *V*_rev_K^+^ (see below). Smaller effects are produced in the presence of a reduced shift in *V*_rev_K^+^ (*e.g.* Fig. 1B, C).

**Fig. 2:**
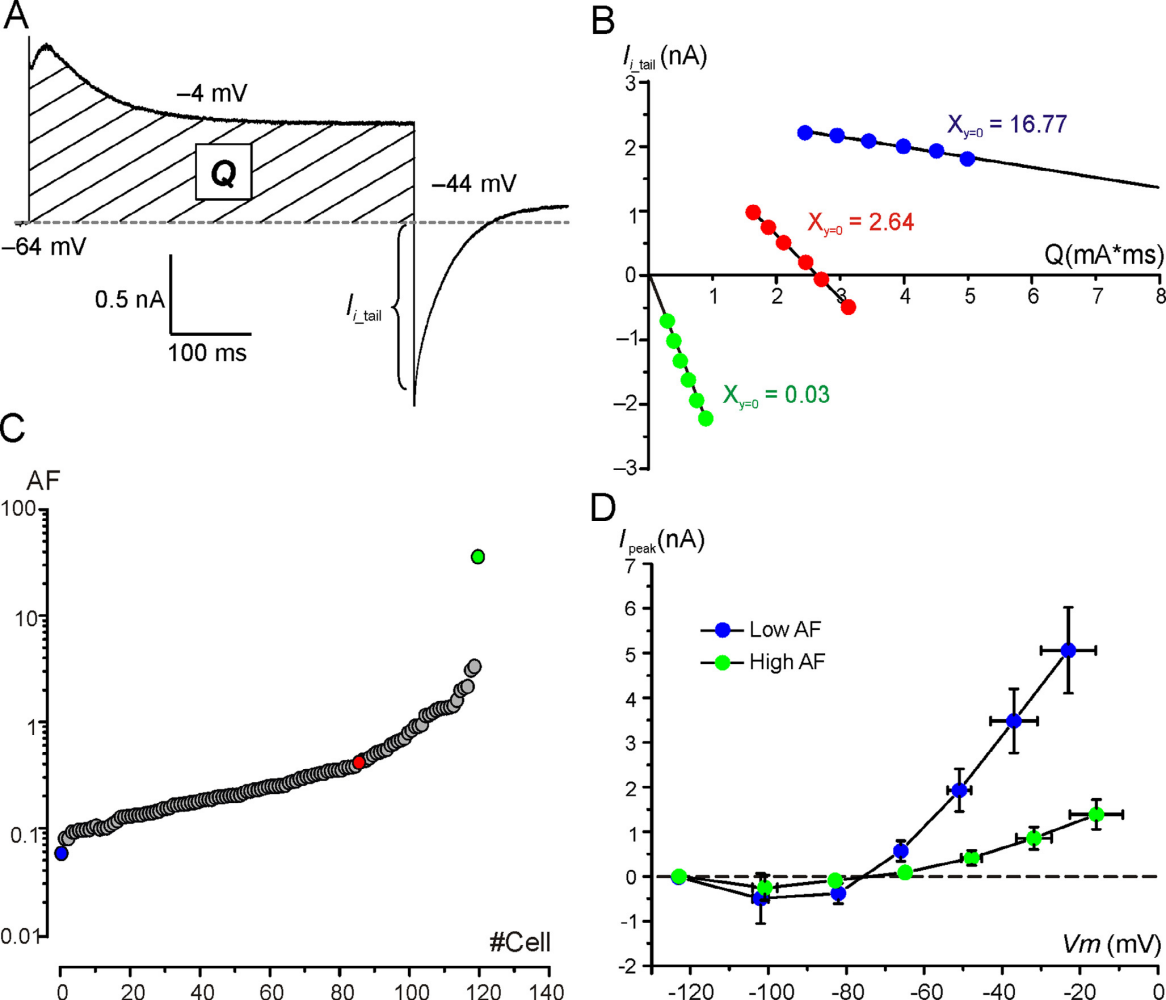
K^+^ accumulation varies largely between hair cells. (**A**) Example current trace showing the outward current which was integrated (dashed area) to calculate *Q* (see Experimental procedures). (**B**) Representative *I_i_*__tail_ – *Q* relations (see text) for the cell responses shown in Fig. 1(**A**) (green filled circles), (**B**) (red filled circles) and (**C**) (blue filled circles). V_conds_ varied between −14 mV and 36 mV (10 mV step increment). The numerical values refer to the intercept of the linear fit extrapolation with the X-axis. (**C**) Accumulation Factor (AF) values for all cells investigated (*n* = 120) − each circle represents a cell. AF varied by three orders of magnitude between cells (note the semi-logarithmic scale). The green, red and blue filled circles refer to the cell responses shown in Fig. 1(**A–C**), respectively. All recordings were obtained at RT. (**D**) Mean steady-state current(*I*_steady_)-voltage relation for low- and high-AF cells. *V*_m_: membrane voltage. Voltages were corrected for *V*_Rs_ and currents were subtracted for leakage. The raw data for all 120 recordings and the Origin files can be found in folder *NeuroscienceFig2*. Analysis in *NeuroscienceDatasheetExcel*. (For interpretation of the references to colour in this figure legend, the reader is referred to the web version of this article.)

In principle, one may expect that larger K^+^ currents will produce larger shifts of *V*_rev_K^+^. Clearly, this was not the case for the examples shown in Fig. 1, C. The major problem in correlating any change in *V*_rev_K^+^ with K^+^ currents properties was that they varied largely between recordings. Therefore, we first normalized the shift of *V*_rev_K^+^ to K^+^ efflux by plotting tail K^+^ currents as a function of the quantity of K^+^ ions (quantity of charge, *Q*; see Materials and Methods) exiting the hair cell in response to different V_conds_ (usually from −14 mV to 36 mV). Fig. 2A illustrates the procedure for calculating *Q* at a single V_cond_ (same cell as Fig. 1A, V_cond_: −4 mV). The *I*_i_tail_/*Q* relationships for the current responses of Fig. 1A, B and C are shown in Fig. 2B. Data points were well fitted by a linear function, the extrapolation of which to the x-axis provided the quantity of K^+^ ions theoretically required to reverse *I*_i_tail_ at −44 mV. The progressively steeper *I*_i_tail_/*Q* relationship (green filled circles *vs*. red filled circles *vs*. blue filled circles in Fig. 2B) indicates a progressively higher sensitivity of *V*_rev_K^+^ to K^+^ efflux for the cell response shown in Fig. 1A compared to those of Fig. 1B, C. Moreover, negative *I*_i_tail_ values (Fig. 2B, green and red filled circles) indicate that *V*_rev_K^+^ was shifted above −44 mV during V_cond_.

The reciprocal value of the intercept was calculated for all investigated Type I hair cells (*n* = 120; Fig. 2C), which was named “accumulation factor” (AF), since it provides an estimate of the K^+^ efflux required to shift *V*_rev_K^+^ to −44 mV. A large AF means that even a small K^+^ efflux can shift *V*_rev_K^+^ substantially.

Paradoxically, the outward K^+^ currents in cells with a large AF were smaller than in those with a small AF (note that the outward current in Fig. 1A is much smaller than that in Fig. 1C). Following subtraction of the leak current as calculated between −124 mV and −114 mV (see Methods), comparison of the two groups of 10 cells of similar age with the smallest or the largest AF revealed that the former had significantly larger peak outward K^+^ currents (5.44 ± 1.05nA at −24 ± 8 mV; PD: 15.56 ± 5.53; *n* = 10; median: 5.53 nA) compared to the latter ones (2.04 ± 0.52 nA at −22 ± 9 mV; PD: 16.40 ± 2.32; *n* = 10; median: 2.59 nA) – (*t*(13.17) = 9.11, *p* < 0.0001; *F*(9,9) = 4.07, *p* = 0.048; unpaired *t*-test with Welch correction). The smaller peak outward K^+^ current amplitude seen in high-AF than in low-AF cells was not necessarily due to a smaller *G*_K,L_ amplitude, as it could be also explained by a smaller driving force for K^+^ to exit because of substantial intercellular K^+^ accumulation. Consistent with this hypothesis, AF correlated with a significantly more depolarized *V*_rest_ of the cells. *V*_rest_ was −74.4 ± 2.9 mV (*n* = 9; median: −75 mV) in low-AF cells *vs*. −70.1 ± 3.5 mV (*n* = 10; median: −71 mV) in high-AF cells – (*t*(16) = 2.76, *p* = 0.014; *F*(9,7) = 1.44, *p* = 0.6446; unpaired *t*-test). Since a small *I*_h_ was only detected in one low-AF cells and one high-AF cells (not shown), and *R*_m_ between −124 mV and −114 mV was not significantly different between cells with a low-AF cells (1.48 ± 1.66 GΩ; *n* = 10; median: 1.11 GΩ) and a high-AF (1.54 ± 1.25 GΩ; *n* = 10; median: 1.10 GΩ) – (U(100,110) = 45, *p* = 0.7245; Mann–Whitney Test), the more depolarized *V*_rest_ in high-AF cells was not attributable to *I*_h_ or to “leaky” hair cells. Therefore, the above results indicates that in high-AF cell responses, intercellular K^+^ accumulation already occurs at rest.

The difference in the steady-state outward K^+^ current amplitude between low- and high-AF cells (Fig. 2D) was even larger than the difference in the peak outward K^+^ currents. For example, the mean steady-state outward K^+^ current was 4.28 ± 0.83 nA at −31 ± 6 mV; *n* = 10; median: 4.51 nA in low-AF cells compared to 0.84 ± 0.25 nA at −32 ± 4 mV; *n* = 10; median: 0.88 nA) – (*t*(12.85) = 10.62, *p* < 0.0001; *F*(9,9) = 11.05, *p* = 0.0014; unpaired *t*-test with Welch correction).

The above results indicate a stronger decrease of the outward K^+^ current during step depolarization in high-AF cells compared to low-AF cells. For example, at −31 ± 6 mV the outward current in low-AF cells relaxed to 96 ± 3% of the peak current (*n* = 10; median: 97.79%), while it relaxed to 64 ± 14 % at −32 ± 4 mV in high-AF cells (*n* = 10; median: 67.74%) − (*t*(10.08) = 7.32, *p* < 0.0001; *F*(9,9) = 16.63, *p* = 0.0003; unpaired *t*-test with Welch correction). This result is consistent with outward K^+^ current relaxation in high-AF cells mainly produced by progressive intercellular K^+^ accumulation during V_cond_. The role of voltage-dependent inactivation should instead be minimal since even in low-AF cells the small outward current relaxation was associated to a shift of *V*_rev_K^+^ towards depolarized voltages (*e.g.* Figs. 1C and 2B blue filled circles).

#### But what determines the value of AF?

It is unlikely that the integrity of the sensory epithelium is responsible because large AF values were found both *in situ* and in dissociated hair cells (the largest AF was in fact observed for a dissociated cell; Fig. 1A). Along with the experiments, we also found hair cells with a large AF despite the impression that, during the “cleaning” procedure (see Experimental procedures), the attached calyx had been removed, as previously described (Spaiardi et al., 2017). One possibility is that only the calyx *outer* membrane was removed during the above experiments and as such K^+^ was effectively confined in the intercellular space by the sole calyx *inner* membrane, which is not visible by optical microscopy. For this to occur, however, the K^+^ channels expressed in the calyx inner membrane have to close following removal of the calyx outer membrane, otherwise K^+^ would simply leak into the extracellular space. Indeed, rapid run-down of ion channels activity after patch excision is not unusual (Becq, 1996, Jospin et al., 2002). An alternative possibility is that following damage to the calyx, the inner and outer membranes flatten against each other. Following seal formation with the residual calyx membrane, the latter might have been sucked into the patch pipette together with the hair cell membrane before breaking into the hair cell. These scenarios would all be consistent with a preserved synaptic cleft in high-AF cells. The continuous changes of AF along all cells recorded, therefore, most likely reflect a virtual “infinite” range of experimental conditions in terms of damage to the calyx.

### Intercellular K^+^ removal

Following its accumulation during depolarization, intercellular [K^+^] returned to a lower value upon repolarization (Fig. 1A, gray arrow). Potassium can exit the synaptic cleft through pre- and post-synaptic ion channels/active transporters, and by simple aqueous diffusion towards the interstitial (bath) solution. The latter possibility seems of minor importance in hair cells with high-AF, since simple diffusion was unable to compensate even for the small *I*_K,L_ amplitude at −64 mV (*V*_rest_ was significantly more depolarized than that in low-AF cells). Since *G*_K,L_ does not inactivate nor deactivate significantly in the range of the hair cells receptor potential (Spaiardi et al., 2017), it behaves like a large linear conductance through which K^+^ can flow in either direction depending on the driving force, suggesting it might have a primary role not only in intercellular K^+^ accumulation but also in its clearance. To investigate this possibility in more detail, we looked for any difference in the inward current through *G*_K,L_ between low-AF and high-AF cells. Previous findings have shown that *G*_K,L_ deactivation kinetics appeared faster in hair cell recordings with strong K^+^ accumulation (Spaiardi et al., 2017), which is similar to the current responses shown in Fig. 1 (cyan traces). If intercellular K^+^ can increase during outward K^+^ current elicited by depolarization, it is likely to decrease during inward K^+^ currents elicited by hyperpolarization. In the latter case, the shift of *V*_rev_K^+^ toward negative voltages would concomitantly reduce the driving force for K^+^ to enter the cell, thus producing an apparent acceleration of *G*_K,L_ deactivation time course (Spaiardi et al., 2017). Having defined AF as an index of the residual calyx “quality”, we compared the time required for the instantaneous inward current at −124 mV to decrease by 90% (t-90%) in low- and high-AF cells. We chose t-90% instead of the decay time constant because the complex deactivation time course of *G*_K,L_ requires more than one exponential to be fitted (Spaiardi et al., 2017). We found that, on average, *G*_K,L_ deactivated faster in high-AF than in low-AF cells, consistent with K^+^ clearance by the inward *I*_K,L_ in the presence of a more intact residual calyx. t-90% was 82.37 ms (±75.29 ms; *n* = 10; median: 67.58) in high-AF cell compared to 101.41 ms (±61.64 ms; *n* = 9; median: 94.82 ms) in low-AF cells – (*t*(17) = 0.5986, *p* = 0.5573; (*F*(1.492, 9,8); *p* = 0.5839; Unpaired *t*-test). However, the difference was not statistically significant, possibly because the gating of *G*_K,L_ is affected not only by membrane voltage but also by K^+^ since it is slowed down by an increase of external K^+^ concentration (Contini et al., 2012, Contini et al., 2017). Thus, the residual calyceal cleft produces two opposed effects, whereby *G*_K,L_ deactivation kinetics is slowed down by the increased K^+^, but accelerated by K^+^ clearance.

The hypothesis that *G*_K,L_ is involved in intercellular K^+^ clearance is also supported by experiments in which Cs^+^ was used instead of K^+^ in the pipette (Intra_Cs^+^). Different from most voltage-gated K^+^ conductances, *G*_K,L_ is significantly permeable to Cs^+^ (Rennie and Correia, 2000). In the presence of Intra_Cs^+^, a substantial current could be recorded at all membrane voltages (see Table 1 for a comparison with Intra_K^+^). However, *G*_K,L_ is less permeable to Cs^+^ than K^+^, causing the macroscopic current to reverse at significantly more depolarized voltages (−40.0 ± 6.3 mV; *n* = 17) than with Intra_K^+^ (near −74 mV, see above). This depolarized *V*_rev_ is well consistent with the estimated *V*_rev_ of −39 mV in our experimental condition, as calculated by Eq. (2), given a total [Cs^+^] in the pipette solution of 88 mM and a total [K^+^] in the extracellular solution of 5.8 mM, and given the reported permeability ratio of Cs^+^ to K^+^ of 0.31 (Rüsch and Eatock, 1996). However, a more recent study showed a permeability ratio of Cs^+^ to K^+^ of 0.15 (Wong et al., 2004), which in our experimental conditions would give a *V*_rev_ of −21 mV. The different permeability values reported might have been caused by intercellular ion accumulation/depletion (see below).

**Fig. 3:**
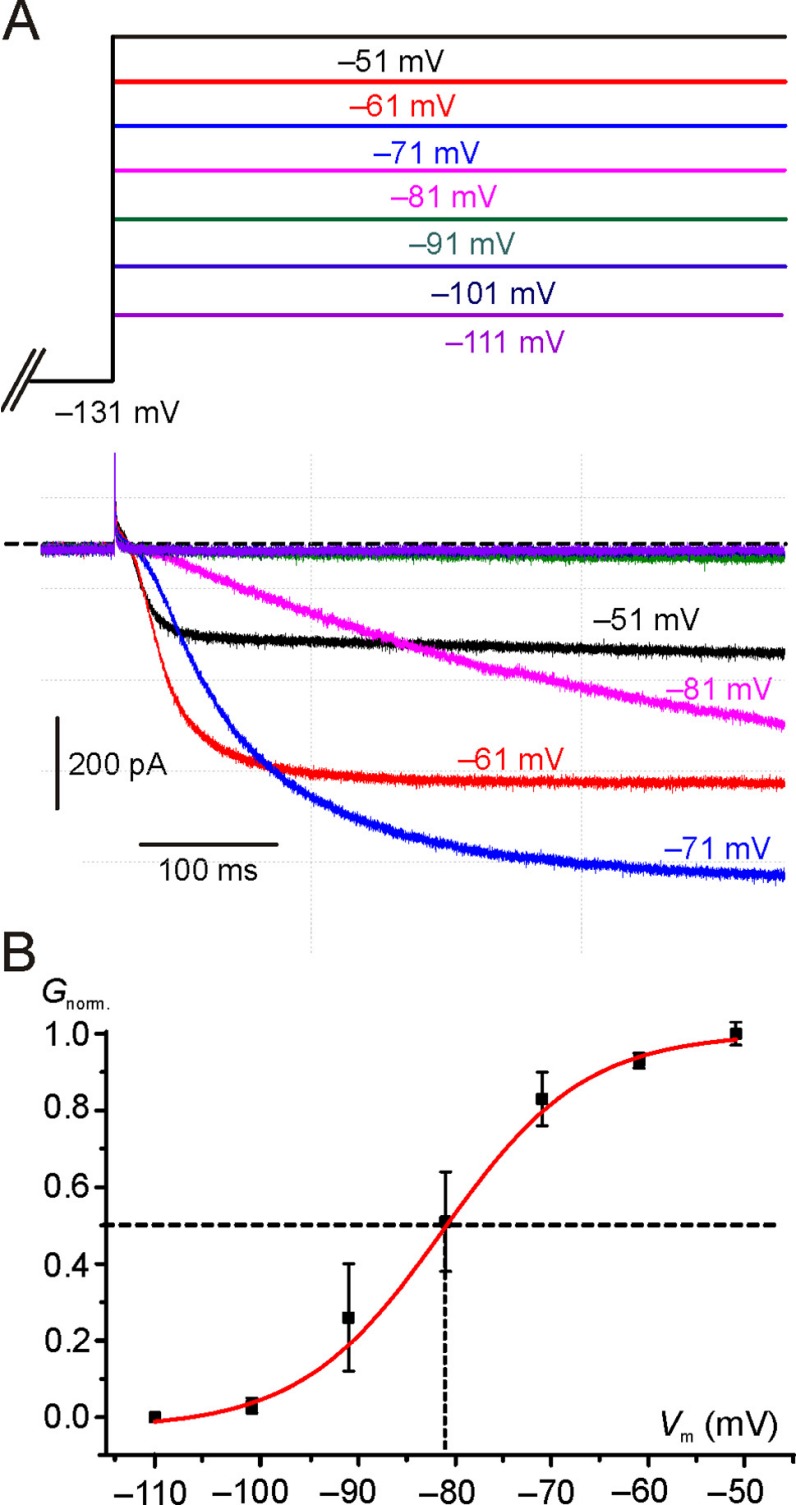
*G*_K,L_ activation curve in Intra_Cs^+^. (**A**) Representative macroscopic currents recorded from a Type I hair cell with Cs^+^ instead of K^+^ in the pipette solution (Intra_Cs^+^). The cell was conditioned at −131 mV for 500 ms (only the last portion of the trace is shown) and then depolarized for 500 ms to different V_tests_ as shown at the top. The horizontal dashed line indicates the zero current level. Hair cell in situ, P50, RT. *R*_s_: 3.6 MΩ.File: 19522005. (**B**) Mean (±S.E.) normalized activation curve (*G*_norm._) obtained by calculating the chord conductance from the current amplitude at the end of the 500 ms. The red line indicates fitting by Eq. (3). Mean *V*_1/2_: −81.2 ± 2.5 mV (*n* = 3); mean *S* 7.3 ± 1.5 (*n* = 3). Files: 17629020; 17703001; 19522005. See folder *NeuroscienceFig3* for raw data and Origin files. Analysis can be found in *NeuroscienceDatasheetExcel*. (For interpretation of the references to colour in this figure legend, the reader is referred to the web version of this article.)

Given the depolarized *V*_rev_ in Intra_Cs^+^, the current was inward in the voltage-activation range of *G*_K,L_ (Fig. 3A). The mean normalized *G*_K,L_ activation curve obtained by 3 Type I hair cells is shown in Fig. 3B. *G*_K,L_ activation curve was obtained by fitting with Eq. (3) (red line) the normalized chord conductance, calculated from the current elicited at each voltage and considering a *V*_rev_ of −40 mV. Please note that the reversal potential is not actually fixed at −40 mV, as it will change depending upon ion accumulation or clearance in the synaptic cleft. However, these experiments were only aimed at investigating the voltage range of *G*_K,L_ activation with Cs^+^ as the ion current carrier, and not its precise value at each voltage. Like with Intra_K^+^ (Rennie and Correia, 1994, Rüsch and Eatock, 1996, Spaiardi et al., 2017), *G*_K,L_ started activating close to −100 mV. Because of the very slow activation kinetics of *G*_K,L_ at hyperpolarized voltages, several seconds are required to reach a steady-state (Spaiardi et al., 2017) – note that *G*_K,L_ is still increasing at the end of the 500-ms voltage step to −81 mV (Fig. 3A; magenta trace). Therefore, Fig. 3B does not describe the steady-state *G*_K,L_ activation curve but, again, it is meant to show its low-voltage activation threshold in Intra_Cs^+^.

**Fig. 4:**
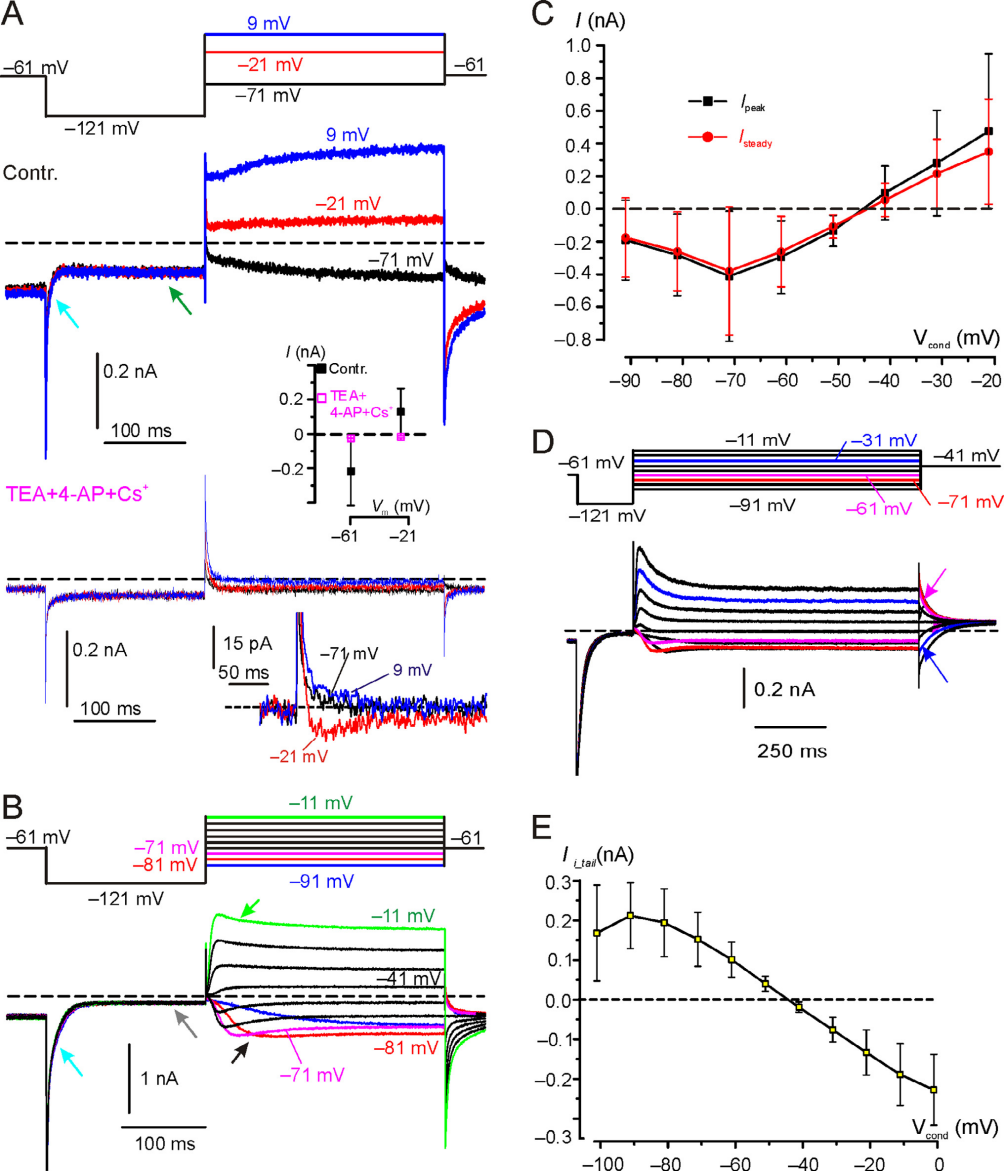
Current recorded from Type I hair cells with Intra_Cs^+^. (**A**) The cell was held at −61 mV and then conditioned at −121 mV to deactivate *G*_K,L_ prior to stepping at different V_tests_ as shown in the voltage protocol at the top. The top panel shows the current response in control (Contr.) conditions. Upon conditioning at −121 mV, *I*_K,L_ rapidly and completely deactivated (cyan arrow), while *I*_h_ slowly activates (green arrow). Following depolarization, *I*_K,L_ activation time course can be seen at −71 mV, at which potential *G*_K,L_ activation kinetics are rather slow (Spaiardi et al., 2017). The bottom panel shows the current response in the same cell, after perfusion with the extracellular solution containing TEA, 4-AP and Cs^+^. The inward and outward currents were substantially reduced due to the block of *G*_K,L_(and *G*_h_ at −121 mV). The small inward current at −21 mV is consistent with Ca^2+^ influx through voltage-gated Ca^2+^ channels. The average (±SD) effect of TEA + 4-AP + Cs^+^ upon the current elicited at −61 mV and −21 mV is shown in the inset (SD for the response in magenta is smaller than the symbol). A selected portion of the currents recorded in TEA + 4-AP + Cs^+^ after leakage subtraction is also shown at the bottom. *In situ*, BT, P18. *R*_s_: 8.5 MΩ. Files: 17626015 & 17626016 (see *NeuroscienceFig4* for raw data and Origin file). (**B**) Macroscopic currents from a different Type I hair cell, showing clear inward and outward current relaxation. The exponential decrease of the inward current (cyan arrow) at −121 mV corresponds to *G*_K,L_ deactivation time course. The steady-state inward current at −121 mV was close to zero (gray arrow), consistent with full deactivation of *G*_K,L_ and little, if any, *I*_h_ in this cell. The black and green arrows indicate relaxation of inward and outward currents, respectively. *In situ*, BT, P15. *R*_s_: 12.8 MΩ. File: 17614024 (see *NeuroscienceFig4* for raw data and Origin file). (**C**) Average (*n* = 9) peak and steady-state current–voltage relation. (**D**) Macroscopic currents elicited by prolonged V_conds_. From V_hold_ of −61 mV the cell was conditioned at −121 mV for 200 ms, then stepped at different V_tests_ for 1,000 ms, and finally stepped to −41 mV. *In situ*, RT, P47. *R*_s_: 4.91 MΩ. File 19513016. RT. (**E**) Mean (*n* = 3) *I*_i_tail_ measured at −41 mV as a function of V_cond_. All recordings *in situ* at RT. Files 19510009 (P44. Rs: 4.70 MΩ), 19513003 (P47, *R*_s_: 4.36 MΩ) and 19513016 P47, Rs: 4.91 MΩ). (For interpretation of the references to colour in this figure legend, the reader is referred to the web version of this article.)

On average, the steady-state inward current at −61 mV was −0.28 ± 0.22nA (*n* = 16). This inward current should be almost exclusively carried by K^+^ through *G*_K,L_ since Na^+^ does not permeate *G*_K,L_ (Rennie and Correia, 2000). Moreover, a significant contribution from *G*_h_ can be excluded since it generally activates at voltages more negative than −60 mV (Maccaferri et al., 1993), as also shown in mouse Type I hair cells (Horwitz et al., 2011). Finally, the voltage-gated Na^+^ current (*I*_Na_) is absent in postnatal mouse Type I hair cells (Géléoc et al., 2004), and the voltage-dependent Ca^2+^ current (*I*_Ca_) expressed by mouse Type I hair cells activates positive to −60 mV and is very small (Dulon et al., 2009). In agreement with the above reports, perfusion with an extracellular solution containing TEA, 4-AP and Cs^+^ (see Experimental procedures for solution composition) confirmed the absence of *I*_Na_ and the presence of a very small *I*_Ca_ (Fig. 4A, bottom traces). The decrease of the sustained inward current at V_hold_ of −61 mV and of the instantaneous inward current at −121 mV (Fig. 4A) is consistent with the block of *G*_K,L_ by millimolar 4-AP (Rennie and Correia, 1994). The reduction of the sustained current at −121 mV is likely due to the block of *I*_h_ by Cs^+^, consistent with Meredith et al. (2012) where 1–5 mM external Cs^+^ completely blocked *I*_h_ in gerbil *crista* Type I hair cells. As far as the nature of the residual current is concerned, fitting of the inward current measured at −121 mV (−95 ± 13 pA; *n* = 3), −91 mV (−34 ± 4 pA; *n* = 3), −81 mV (−31 ± 3 pA; *n* = 3), −71 mV (−25 ± 4 pA; *n* = 3) and −61 mV (−25 ± 4 pA; *n* = 3) gave a reversal potential of +8 mV, which was considered to be mostly leak current. Assuming that leakage did not change before and after perfusion of TEA, 4-AP and Cs^+^, its contribution to the control current at −61 mV (−218 ± 200 pA; *n* = 3) was therefore 11%.

The inset in the bottom panel of Fig. 4A shows *I*_Ca_ (red trace) after leak current subtraction.

Perfusion of TEA, 4-AP and Cs^+^ also blocked the outward Cs^+^ currents elicited by voltage steps less negative than *V*_rev_ (Fig. 4A). For example, at −21 mV the current changed from 129 ± 135 pA (*n* = 3) to −18 ± 7 pA (*n* = 3) − see inset in Fig. 4A.

In a subset of 9 cells recorded with Intra_Cs^+^ we analysed the activation kinetics of *G*_K,L_ at voltages negative to −61 mV to avoid overlap with *G*_K,v_, which activates positive to −50 mV (Rennie and Correia, 1994, Rüsch and Eatock, 1996, Spaiardi et al., 2017). In the example reported in Fig. 4B, the inward currents recorded between −71 mV and −51 mV showed a clear relaxation (black arrow) during the 300 ms V_test_ duration. Since at these hyperpolarized voltages *G*_K,L_ does not inactivate (Spaiardi et al., 2017), while *I*_Na_, *I*_Ca_ and (at least at −61 mV) *I*_h_ are not activated, inward current relaxation can only be explained by a progressive leftward (towards more negative voltages) shift of *V*_rev_ as produced by removal of intercellular K^+^. The degree of inward current relaxation, measured as the difference between the peak and the steady-state inward current amplitude (Fig. 4C), varied substantially between cells. For example, no inward current relaxation was visible in the current response shown in Fig. 4A (and Fig. 3A above). Presumably, as for intercellular K^+^ accumulation, the “quality” of the residual calyx is also important for K^+^ clearance, as free diffusion of K^+^ into the cleft from the bath will rapidly substitute K^+^ entering the hair cell, thus minimizing the shift of *V*_rev_. On average, during the 300 ms V_cond_ of −61 mV, the inward current decreased to 87.90 ± 11.74% (*n* = 9) of the initial peak value. In the same cells, voltages less negative than *V*_rev_ elicited outward currents showing variable degree of relaxation (*e.g.*, green arrow in Fig. 4B). On average, during 300 ms V_cond_ of −21 mV, the outward current relaxed to 77.44 ± 13.99 % (*n* = 9) of the initial peak value. Outward current relaxation is consistent with intercellular Cs^+^ accumulation in a similar way as K^+^ (Fig. 1A). The mean current–voltage relations for the peak and steady-state outward currents are shown in Fig. 4C. A weak correlation (Pearson correlation coefficient (*R*): 0.31; *p*: 0.42) was found between the percentage of inward current relaxation at −61 mV and of outward current relaxation at −21 mV by linear regression of data, possibly because inward current relaxation overlaps to *G*_K,L_ activation time course which at hyperpolarized voltages is very slow (time constant >100 ms at −64 mV; Spaiardi et al., 2017).

To further test for intercellular K^+^ clearance by *G*_K,L_, we investigated whether large inward currents could produce reversal of *I*_i_tail_ from inward to outward (*i.e.* in the opposite direction compared to *I*_i_tail_ reversal produced by outward K^+^ currents; Fig. 1A). We showed previously that increasing the duration of a conditioning depolarizing pulse increased intercellular K^+^ accumulation (Contini et al., 2012; Fig. 2). To ease detection of *I*_i_tail_ reversal, *I*_i_tail_ was tested at V_test_ of −41 mV, close to the average *V*_rev_ of −40 mV, and V_cond_ duration was prolonged to 1000 ms. As shown in the cell response of Fig. 4D, *I*_i_tail_ following sustained outward currents (*e.g.* blue trace at −31 mV) was inward, consistent with intercellular Cs^+^ accumulation. However, following long-lasting inward currents (*e.g.* at −61 mV, magenta trace in Fig. 4D), *I*_i_tail_ reversed to outward, consistent with intercellular K^+^ clearance by *G*_K,L_ (the only active conductance at −60 mV). The mean *I*_i_tail_/V_cond_ relation, obtained from three cells by using the voltage protocol illustrated above, is shown in Fig. 4E.

Taken as a whole, the above experiments show that *G*_K,L_ is responsible for intercellular K^+^ accumulation *or* clearance depending upon hair cell depolarization *or* hyperpolarization, respectively. It should be noted that, depending upon *V*_rev_K^+^, *G*_K,v_ is also contributing to K^+^ flux into the cleft during hair cell depolarization above −40 mV, and back into the hair cell during hair cell hyperpolarization before it deactivates.

### Voltage-gated K^+^ channels at the calyx

In a previous study, we found that large outward K^+^ currents evoked by calyx depolarization could produce a shift of *V*_rev_K^+^ toward less negative voltages (see Fig. 7C in Contini et al., 2012), consistent with K^+^ accumulation in the synaptic cleft due to K^+^ exit through voltage-gated K^+^ channels expressed at the calyx *inner* membrane. By double-patching the Type I hair cell and the associated calyx in an *in situ* turtle *crista* preparation, Contini et al. (2017) showed that elevation of K^+^ in the synaptic cleft could result from depolarization of either the presynaptic hair cell or the associated postsynaptic calyx. Immunolabelling studies have reported the expression, at the rodent calyx *inner* membrane, of voltage-gated K^+^ channel subunits K_V_1, K_V_7 and K_V_11 (Sousa et al., 2009, Lysakowski et al., 2011, Spitzmaul et al., 2013, Holt et al., 2017), while the calyx outer membrane expressed K_V_7 and K_V_11, but not K_V_1 subunits (Lysakowski et al., 2011). Since the permeability to Cs^+^ is large for K_V_11 channels (Zhang et al., 2003, Youm et al., 2004), but very low for K_V_1 and K_V_7 channels (Chao et al., 2010, Cloues and Marrion, 1996), to get more information about the channels responsible for K^+^ flux across the calyx inner membrane, we recorded from the calyx with Intra_Cs^+^ in the pipette and administered K^+^ channel blockers by a local perfusion pipette.

**Fig. 5:**
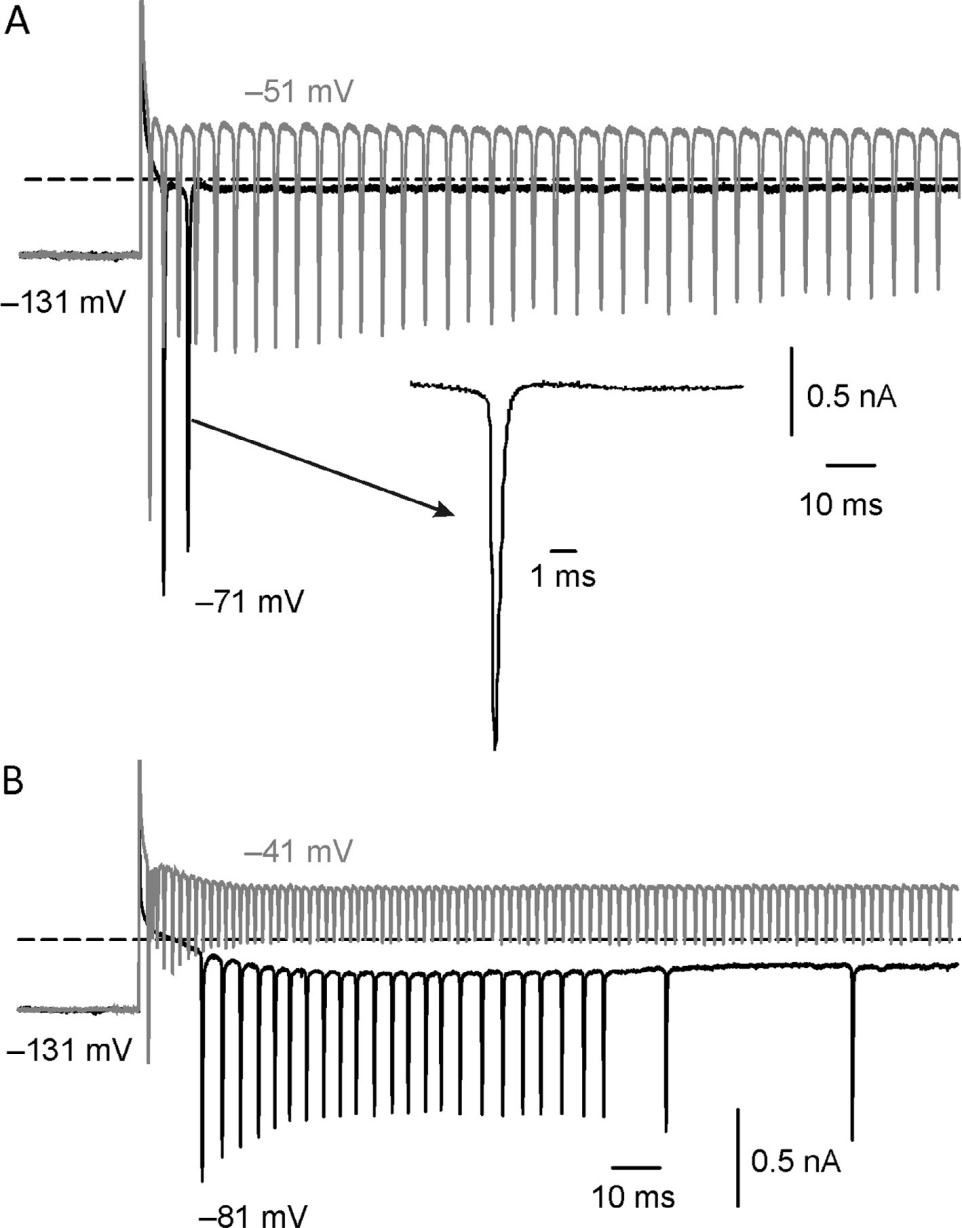
Repetitive action Na^+^ currents recorded from calyces with Intra_Cs^+^. (**A**) Whole-cell currents recorded from a calyx in response to the voltage steps shown next to each trace, delivered from a V_cond_ of −131 mV. Two action Na^+^ currents were elicited at −71 mV, while further depolarization evoked a repetitive discharge. The 2nd action Na^+^ current elicited at −71 mV is also shown at larger time resolution (arrow). BT; P16. File: 17728005. (**B**) Whole-cell current recorded from another calyx, delivered from a V_cond_ of −131 mV, showing repetitive discharge of Na^+^ currents already at −81 mV. *In situ*, BT, P19. File: 17712026. See *NeuroscienceFig5* for raw data and Origin files.

In most calyces (16 out of 20), depolarization above −71 mV elicited a volley of rapid transient inward currents, whose frequency increased with depolarization (Fig. 5A). Since no EPSCs were detected, transient inward currents presumably reflected action potentials generated at the axon encoder (the spike trigger zone) escaping voltage-clamp (Williams and Mitchell, 2008), *i.e.* action Na^+^ currents (Na^+^ currents during action potential generation). Analogous space-clamp problems have been reported with a K^+^-based intracellular solution in whole mount vestibular preparations (Contini et al., 2017, Highstein et al., 2015). In 10 of the 16 calyces showing repetitive firing, action Na^+^ currents could be elicited already at −81 mV (Fig. 5B), indicating that the encoder region was depolarized by intracellular Cs^+^, again because of poor space-clamp conditions (Spruston and Johnston, 2008, Fleidervish and Libman, 2008).

However, in *dissociated* rodent vestibular calyces a single transient Na^+^ current was elicited by depolarization above −60 mV (Hurley et al., 2006, Rennie and Streeter, 2006, Dhawan et al., 2010, Meredith et al., 2011), consistent with good space-clamp of the isolated terminal.

**Fig. 6:**
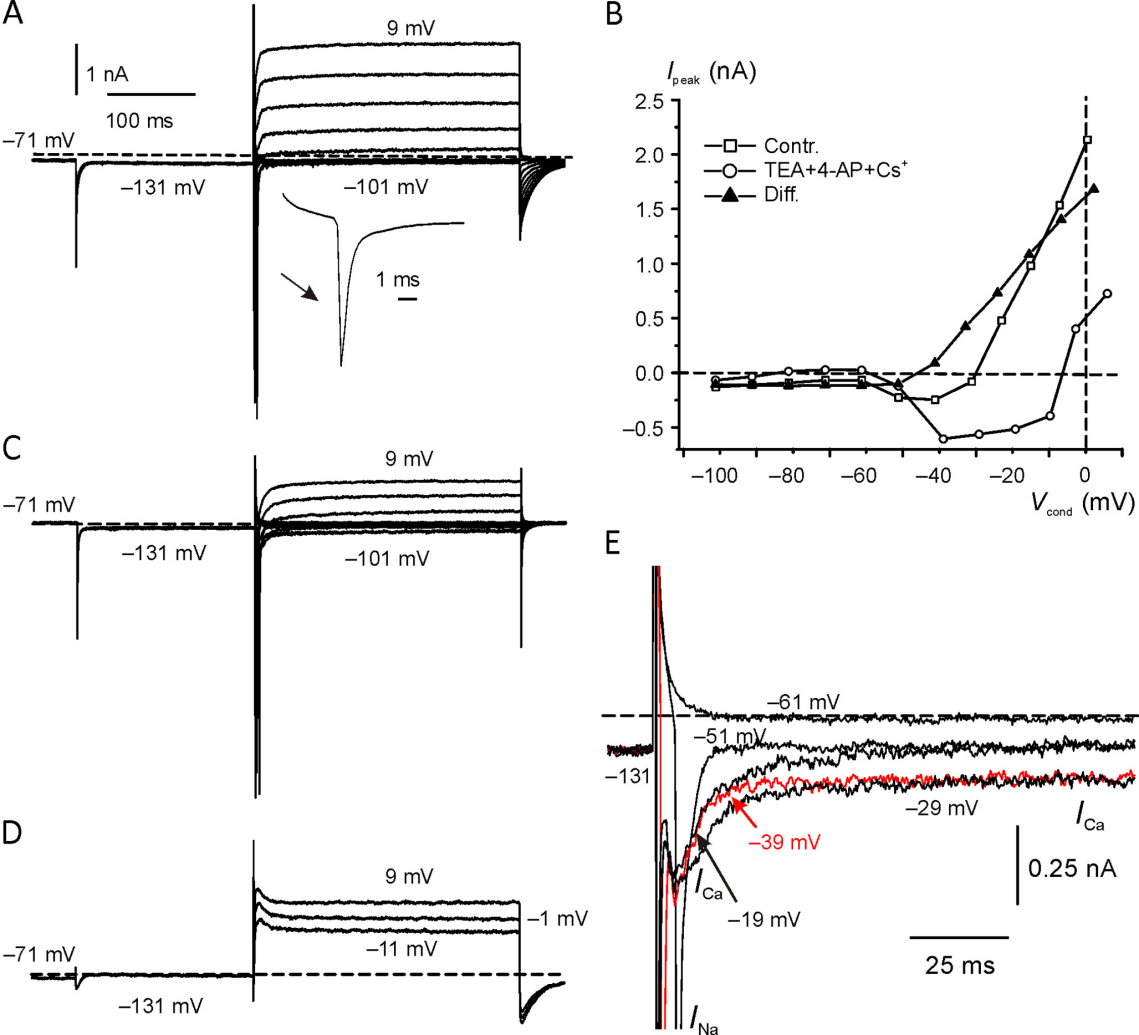
Single action Na^+^ current recorded from a calyx with Intra_Cs^+^. (**A**) Macroscopic currents recorded in response to V_tests_ from −101 mV to 9 mV (10 mV increment), after V_cond_ of −131 mV; V_hold_: −61 mV. *R*_s_: 3.9 MΩ. *In situ*, BT, P15. File: 17623021. The inset shows an expansion of the action Na^+^ current elicited at V_test_ of −51 mV. Vertical and horizontal scale bars also apply to (**C**) and (**D**). (**B**) Current-Voltage relations between *I*_peak_ (after *I*_Na_ peak) and V_cond_. Values have been corrected for voltage drop across *R*_s_. (**C**) Macroscopic currents after perfusion with an extracellular solution containing TEA + 4-AP + Cs^+^. File: 17623023. (**D**) Differential currents at three selected voltages. (**E**) Selected traces on expanded scales to show the calcium (*I*_Ca_) and the Na^+^ (*I*_Na_) current (the peak of *I*_Na_ has been truncated). *In situ*, BT, P15. See *NeuroscienceFig6* for raw data and Origin files.

In order to avoid the problem of poor clamp, we restricted our analysis to those calyces that, like dissociated calyces, showed a single transient Na^+^ current for depolarization above −61 mV (*n* = 4; files 17622009 (P20); 17623021 (P15); 17629002 (P16); 17630019 (P17). The presence of *I*_Na_ is consistent with cell identification as a calyx since in the mouse, different to the rat (Wooltorton et al., 2007), *I*_Na_ is expressed by vestibular hair cells only before birth (Géléoc et al., 2004). Moreover, *C*_m_ of mouse Type I hair cells is typically below 10 pF (5.28 ± 0.15 pF, Vincent et al., 2014), while the mean *C*_m_ in the four recordings considered to be calyces was 39.0 ± 18.1 pF (*n* = 4). An example of calyx response with a single Na^+^ action current is shown in Fig. 6A. At V_hold_ of −71 mV, a small sustained inward current (−64 pA) was present. On average, the sustained inward current at −71 mV was −132 pA (±10 pA; *n* = 4).

The macroscopic current reversed at −55 mV ± 14 mV (*n* = 4). For depolarization above *V*_rev_, the outward current increased linearly (Fig. 6B, squares). In previous studies with a K^+^-based intracellular solution, calyx terminals revealed two main outward rectifying K^+^ current components: a rapidly activating, rapidly inactivating current sensitive to 4-AP, and a slowly activating current sensitive to TEA (Dhawan et al., 2010, Contini et al., 2012, Horwitz et al., 2014). The absence of a transient outward current here could be due to its complete block by intracellular Cs^+^.

Perfusion with the extracellular solution containing the K^+^ channels blockers TEA [30 mM] and 4-AP [15 mM], plus Cs^+^ [5.8 mM] to also block HCN channels, reduced the inward current in the negative voltage range from −101 mV to −51 mV and the outward current at more depolarized voltages (Fig. 6B, open circles; Fig. 6C). Although we did not run voltage protocols aimed at investigating *I*_h_ properties, three of the four calyces clearly showed its presence at V_cond_ of −131 mV. Therefore, the inward current blocked at most negative voltages could have been *I*_h_. However, since *G*_h_ in mouse vestibular primary neurons is fully deactivated at −60 mV (Horwitz et al., 2014), *I*_h_ should not account for the blocked inward current at −61 mV and −51 mV. The latter current was presumably carried by a low-voltage-activated K^+^ conductance, which was blocked by TEA and 4-AP.

**Fig. 7:**
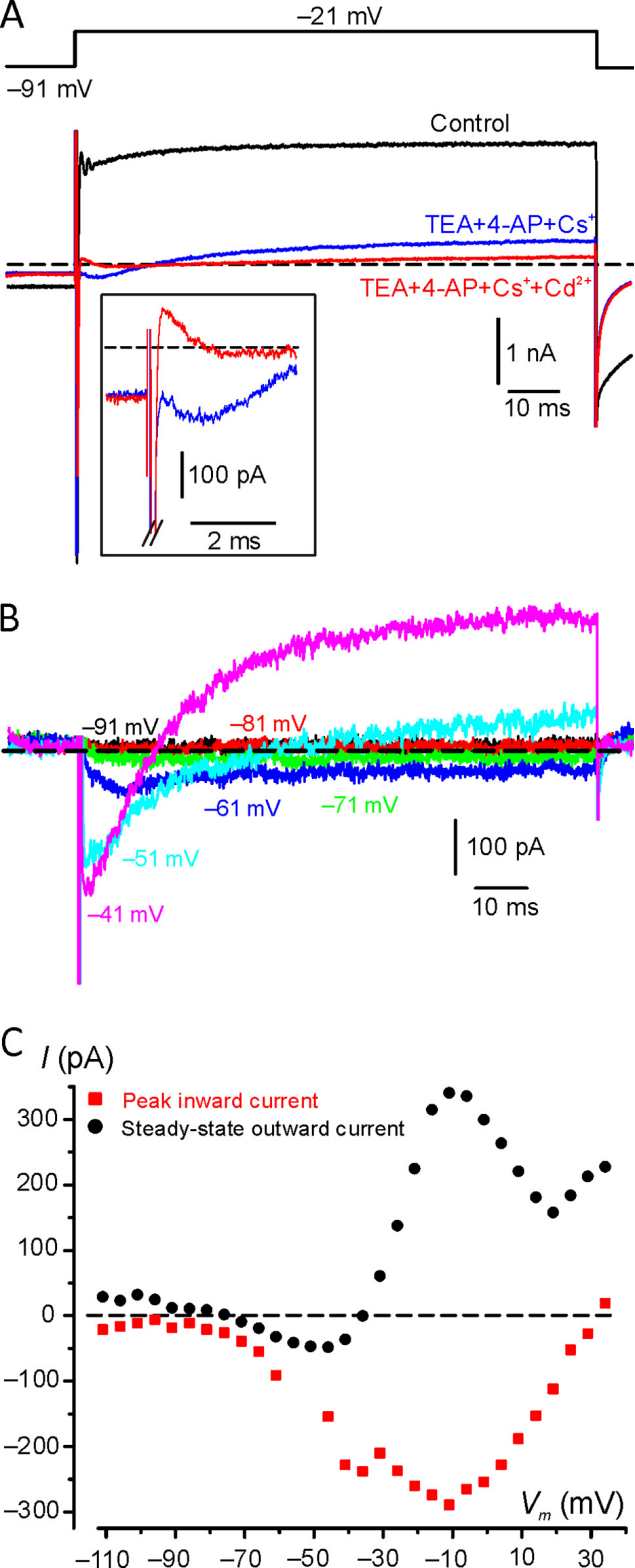
Ca^2+^ and Ca^2+^-dependent K^+^ currents recorded from the calyx. (**A**) Macroscopic currents recorded in response to voltage steps from −91 mV to −21 mV in Extra_std (control condition), TEA + 4-AP + Cs^+^, and TEA + 4-AP + Cs^+^+Cd^2+^. An inward current characterized by a much slower activation time course than *I*_Na_ is unveiled by administration of TEA + 4-AP + Cs^+^, which is blocked by the addition of Cd^2+^ (see inset for expanded time scale; the peak of *I*_Na_ has been truncated). Note that Cd^2+^ also blocked the steady-state outward current. *In situ*, BT, P20, *R*_s_: 6.6 MΩ. Files: 17622009, 17622015 and 17622020. (**B**) Selected traces showing the Cd^2+^-sensitive current (Files 17622015 and 17622020). (**C**) Current-voltage relation for the peak inward and the steady-state Cd^2+^-sensitive current. The peak inward current values at −61 mV and −56 mV are not shown because *I*_Na_ overlap precluded measurement. *In situ*, BT, P20. See *NeuroscienceFig7* for raw data and Origin files.

On average, after delivery of TEA + 4-AP + Cs^+^, the steady-state inward current at −71 mV decreased from −226 pA ± 234 pA (*n* = 4) to −109 ± 134 pA (*n* = 4) and the peak outward current at 9 mV decreased from 3427 ± 1552 pA (*n* = 4) to 1298 ± 0.602 (*n* = 4). The outward current blocked by TEA + 4-AP + Cs^+^, obtained by subtracting the residual current after block from the control current, appeared near −40 mV and increased monotonically with depolarization (Fig. 6D; Fig. 6B, filled triangles). After TEA + 4-AP + Cs^+^ perfusion, another inward current besides *I*_Na_ was clearly detectable in 2 of the 4 cells tested, that activated positive to −61 mV, reached a peak at −39 mV and inactivated partially (Fig. 6E, B, circles). Given its much slower activation kinetics compared to the Na^+^ current, it was likely carried by Ca^2+^. *I*_Ca_ is likely responsible for the apparently less negative activation threshold of the control outward current compared to the blocked current. Voltage-gated Ca^2+^ channels might be functionally associated with the activation of the apamin-sensitive Ca^2+^-dependent K^+^ current (*I*_KCa_) found at the gerbil vestibular calyx terminal (Meredith et al., 2011). Consistent with this hypothesis, in two experiments with stable conditions after TEA + 4-AP + Cs^+^ administration, addition of Cd^2+^ (0.1 mM), which blocks all voltage-gated Ca^2+^ channels at sub-millimolar concentration (Hille, 2001), reduced the steady-state outward current elicited at −21 mV from 341 ± 7 pA (*n* = 2) to 158 ± 40 pA (*n* = 2) (Fig. 7A). Fig. 7B shows the current blocked by Cd^2+^, which was obtained by subtracting the current recorded in TEA + 4-AP + Cs^+^ + Cd^2+^ from that in TEA + 4-AP + Cs^+^. Selected voltages are shown where overlap with *I*_Na_ is minimized. *I*_Ca_ activated near −70 mV and reached a peak at −11 mV. Above −31 mV a slowly developing outward current also appeared, presumably carried by KCa channels. Consistent with the latter hypothesis is the current–voltage relation for the Cd^2+^-sensitive current, measured at the peak and at the steady-state (Fig. 7C). Note the N-shape of the steady-state outward current–voltage relation typical of *I*_KCa_ (Meech and Standen, 1975). To our knowledge, this is the first evidence for the expression of voltage-gated Ca^2+^ channels in the calyx terminal.

## Discussion

Previous studies have shown that outward K^+^ currents elicited in Type I hair cells can produce intercellular K^+^ accumulation, as inferred by the shift in the reversal potential (*V*_rev_K^+^), despite the partial removal of the calyx by the patch pipette (Lim et al., 2011, Contini et al., 2012). The large variability of the shift was attributed to the calyx ‘conditions’, although no correlation was performed because of the difficulty associated with the quantification of calyx damage.

In the present study, after normalization of K^+^ current amplitude, we found a statistically significant difference of *V*_rest_ between Type I hair cells with the largest or the smallest AF. In the same cells, the amplitude of the peak and, even more, of the steady-state outward K^+^ currents elicited by depolarization inversely correlated with AF. In the presence of a barrier to K^+^ diffusion, the above results will assume significance, since intercellular K^+^ accumulation will depolarize *V*_rest_ and reduce the driving force for K^+^ to exit during prolonged hair cell depolarization. Since a large AF could be found despite *putative* calyx removal, however, it seems reasonable to assume that, following damage by the patch pipette, the residual calyx may be too thin to be seen by optical microscopy. An alternative explanation could be that ion accumulation or depletion occurred inside the hair cell (Rennie and Correia, 2000). However, the observed shift of *E*_K_ from −80 mV (as calculated according to Eq. (1)) to −44 mV, would require an increase of the extracellular [K^+^] from 5.8 mM to 24 mM, or a decrease of intracellular K^+^ from 136 mM to 33 mM. The latter possibility seems unlikely given that the hair cell interior is defined by the pipette solution.

A substantial shift of *V*_rev_K^+^ was found in several *in situ* and dissociated Type I hair cells (the largest AF was in fact found in a dissociated cell, Fig. 1A), suggesting that the calyx *inner* membrane is tenaciously attached to the hair cell. It is also possible that a complete removal of the calyx might have never been obtained in our recordings since even in the lowest AF cells we could detect some degree of intercellular K^+^ accumulation (*e*.*g*. Fig. 1C). The above observation is consistent with the abundant presence of intercellular proteins joining the pre- and postsynaptic membranes, which resembles the organization of the septate-like junction (Sousa et al., 2009), a structure involved in restricting K^+^ diffusion at paranodes of myelinated axons (Salzer, 2003, Rosenbluth, 2009). AF may thus represent a valid indicator for the presence of a residual calyx membrane and its influence upon hair cell properties.

Intercellular K^+^ accumulation at rest, and its clearance by inward current through *G*_K,L_ (Fig. 1, Fig. 4), indicate that the calyx *inner* membrane severely restricts aqueous diffusion of ions to and from the bath. This diffusion is likely to be even more restricted in the *in vivo* undamaged calyx. Therefore, [K^+^] in the cleft will critically depend on pre- and postsynaptic K^+^ permeable channels *and* active transports. Na^+^,K^+^-ATPase α-subunits have been detected in rat Type I hair cells and calyx inner and outer membrane (Schuth et al., 2014), which should, at least in principle, be very efficient in preventing large changes of the intercellular K^+^ concentration. This suggests that the large changes in intercellular K^+^ found here and other analogous studies *in vitro* (Lim et al., 2011, Contini et al., 2012) might be an experimental artefact caused by the damage produced by the patch pipette to the calyx impairing active transports, and might not be as evident *in vivo*. However, intercellular K^+^ accumulation has been reported in double-patch recording from the apical region of the Type I hair cell and its associated calyx in the turtle (*i.e.*, the calyx was not pierced: Contini et al., 2017). Moreover, since *G*_K,L_ is fully open at *V*_rest_, a sudden change of the depolarizing mechano-transducer (MET) current will produce an almost synchronous change of *I*_K,L_ amplitude and presumably of intercellular K^+^ concentration. Recording from the calyx while mechanically stimulating the associated Type I hair cell in early postnatal (<P9) excised saccule preparations, Songer and Eatock (2013) showed that the calyx membrane potential could be driven despite the absence of glutamate exocytosis and with a very short delay (<0.5 ms). Although the mechanism of fast signal transmission was not identified, K^+^ exit through *G*_K,L_ seems a good candidate.

In summary, the above studies are consistent with active K^+^ transporters not precluding changes in intercellular K^+^, though their regulatory function remains to be determined.

### Ion channels at the calyx

The vestibular calyx expresses several types of ion channels, whose molecular nature and properties have yet to be fully elucidated. *In situ* recordings using slice preparations from the gerbil *crista* have reported the presence of a non-inactivating K^+^ current which was sensitive to dendrotoxin-K, suggesting the contribution of Kv1.1 and/or Kv1.2 channel subunits, and a slowly-inactivating K^+^ current sensitive to margatoxin, indicating the contribution of Kv1.3 and Kv1.6 channel subunits (Meredith et al., 2015).

In isolated rat calyces, linopirdine and XE991, which are selective blockers of K_V_7 channels, blocked a negatively-activating K^+^ current (Hurley et al., 2006).

Our electrophysiological data have demonstrated that with Cs^+^ in the patch pipette, a small current was present at–61 mV, which was blocked by a combination of TEA, 4-AP and Cs^+^ (Fig. 6B).

Since K_V_1 and K_V_7 channels are very sensitive to 4-AP (K_V_1, Al-Sabi et al., 2013) and TEA (K_V_7; Robbins, 2001) and activate near the cell membrane resting potential (Robbins and Tempel, 2012, Jentsch, 2000), our results are consistent with K_V_1 and K_V_7 channel expression. However, since both K_V_1 and K_V_7 channels are little permeable to Cs^+^ (Chao et al., 2010, Cloues and Marrion, 1996), the relatively large size of the Cs^+^ outward currents (Fig. 6A) may suggest that other K^+^ channels are present.

The K_V_11 conductance appears to be a good candidate since K_V_11 channels are very permeable to Cs^+^ (Zhang et al., 2003, Youm et al., 2004) and immunoreactivity for K_V_11 channel subunits has been reported at the rat calyx membrane (Lysakowski et al., 2011). However, K_V_11 channels are functionally inward rectifiers (Bauer and Schwarz, 2001), which is due to their fast inactivation kinetics combined with slow activation, and fast recovery from inactivation combined with slow deactivation (Smith et al., 1996, Vandenberg et al., 2012). The most notable feature of K_V_11 current is an initial “hook” during deactivation current recordings (Shibasaki, 1987). A K^+^ current with the above properties has not been reported in previous calyx recordings, nor in our experiments (Fig. 6).

In addition to voltage-gated K^+^ channels, we found evidence of a calcium-activated K^+^ current (Fig. 7C), as also described previously in gerbil vestibular calyces (Meredith et al., 2011).

By combining the above results with the reported immunoreactivity for K_V_1 and K_V_7 subunits at the rat calyx *inner* membrane (Lysakowski et al., 2011), a scenario is conceivable where intercellular K^+^ variation directly modulates the calyx membrane potential by K_V_1 and K_V_7 channels.

Finally, the HCN channel blocker ZD7288 has been shown to block the inward current at −100 mV in the voltage-clamped calyx during depolarization of the associated turtle hair cell (Contini et al., 2017). The mouse calyces show a predominant expression of HCN2 channel subtypes (Horwitz et al., 2014). HCN2 channels have a very negative voltage range (activation midpoint: −95 mV; Wahl-Schott & Biel, 2009). Therefore, HCN channels, which carry the *I*_h_, might help clear intercellular K^+^ in a restricted voltage range near *V*_rest_.

### The change of *E_K_* around *V_rest_* can produce either calyx depolarization or hyperpolarization

Non-quantal transmission at the Type I hair cell-calyx synapse is faster than quantal vesicle release (Songer and Eatock, 2013), which may be needed for rapid vestibular reflexes (Eatock, 2018). Different mechanisms have been proposed to sustain non-quantal transmission at the Type I hair cell-calyx synapse, *e.g.* electric, ephaptic, or one mediated by either intercellular K^+^ (Goldberg, 1996) or H^+^ (Highstein et al., 2014). As fluorescent dyes do not pass between the Type I hair cell and the calyx (Songer and Eatock, 2013), gap junctions (*i.e.* direct electrical coupling) are not involved. Ephaptic transmission requires a high intercellular resistance (*R*_i_) and an extended apposition of pre- and postsynaptic membranes, such that current flowing through *R*_i_ produces an extracellular potential drop that instantaneously affects the activity of pre- and postsynaptic voltage-gated channels (Vroman et al., 2013). Such morphological requirements appear to be present at the Type I hair cell-calyx synapse, but no experimental evidence is currently available in favour of this mechanism.

Several lines of evidence support the hypothesis that intercellular K^+^ may contribute to non-quantal afferent signalling. *In vitro* experiments in rodents have shown that K^+^ exiting the Type I hair cell can accumulate in the calyceal cleft (Lim et al., 2011, Contini et al., 2012). Immunolabelling has revealed the expression of K^+^ channel subunits at the rat calyx *inner* membrane (Lysakowski et al., 2011), providing a way for direct calyx depolarization by intercellular K^+^ accumulation. In the turtle, depolarization of the hair cell or of the associated calyx affects *V*_rev_K^+^ in the cellular counterpart (Contini et al., 2017), demonstrating direct bidirectional interaction between the pre- and the postsynaptic membrane. Here, we provide evidence for intercellular K^+^ accumulation at around the resting membrane potential, as demonstrated by the depolarized *V*_rest_ of high-AF Type I hair cells. Moreover, the pharmacological and voltage-dependent properties of the macroscopic currents recorded from the calyx (Hurley et al., 2006, Contini et al., 2012, Meredith et al., 2015; present results), are consistent with the expression of a low-voltage activated K^+^ conductance at the calyx *inner* membrane. Thus, the calyx might be depolarized by intercellular K^+^ accumulation already at rest. Finally, we have shown that intercellular K^+^ is removed from the cleft by *G*_K,L_ during hair cell repolarization. The latter result is of particular interest in relation to the finding that inhibitory hair bundle deflection causes the calyx to hyperpolarize below −60 mV (Songer and Eatock, 2013). Given an intracellular K^+^ concentration for the calyx of 163 mM in their recordings (Songer and Eatock, 2013), the intercellular K^+^ concentration would only have to be less than 15 mM for the Nernst K^+^ equilibrium potential across the calyx inner membrane to be more negative than −60 mV. Therefore, it is tempting to speculate that during inhibitory hair bundle deflection *in vivo*, the decrease of *I*_K,L_ causes a relative (compared to rest) decrease of intercellular K^+^ content, thereby increasing the driving force for K^+^ to exit from the calyx into the cleft through K_V_1 and K_V_7 channels expressed at the calyx inner membrane, thus hyperpolarizing the calyx.

As a final consideration, it should be mentioned that the zero-current potential measured with the hair cell in artificial perilymph is likely to be less depolarized than *in vivo* due to the presumably larger MET current through fully functional MET channels and the relatively low endolymphatic Ca^2+^ concentration (20 μM; Fettiplace, 2017). Therefore, *in vivo V*_rest_ might be even more depolarized than found here.
